# High prevalence of curable sexually transmitted infections among pregnant women in a rural county hospital in Kilifi, Kenya

**DOI:** 10.1371/journal.pone.0175166

**Published:** 2017-03-31

**Authors:** Simon Chengo Masha, Elizabeth Wahome, Mario Vaneechoutte, Piet Cools, Tania Crucitti, Eduard J. Sanders

**Affiliations:** 1 Centre for Geographic Medicine Research–Coast, Kenya Medical Research Institute (KEMRI), Kilifi, Kenya; 2 Laboratory Bacteriology Research, Faculty of Medicine and Health Sciences, Ghent University, Ghent, Belgium; 3 Pwani University, Faculty of Pure and Applied Sciences, Department of Biological Sciences, Kilifi, Kenya; 4 HIV/STI Reference Laboratory, Department of Clinical Sciences, Institute of Tropical Medicine, Antwerp, Belgium; 5 Nuffield Department of Medicine, University of Oxford, Headington, United Kingdom; Fred Hutchinson Cancer Research Center, UNITED STATES

## Abstract

**Background:**

Women attending antenatal care (ANC) in resource-limited countries are frequently screened for syphilis and HIV, but rarely for other sexually transmitted infections (STIs). We assessed the prevalence of curable STIs, defined as infection with either *Chlamydia trachomatis or Neisseria gonorrhoeae* or *Trichomonas vaginalis*, from July to September 2015.

**Methods:**

In a cross-sectional study, women attending ANC at the Kilifi County Hospital, Kenya, had a urine sample tested for *C*. *trachomatis/N*. *gonorrhoeae* by GeneXpert^®^ and a vaginal swab for *T*. *vaginalis* by culture. Bacterial vaginosis (BV) was defined as a Nugent score of 7–10 of the Gram stain of a vaginal smear in combination with self-reported vaginal discharge. Genital ulcers were observed during collection of vaginal swabs. All women responded to questions on socio-demographics and sexual health and clinical symptoms of STIs. Predictors for curable STIs were assessed in multivariable logistic regression.

**Results:**

A total of 42/202 (20.8%, 95% confidence interval (CI):15.4–27.0) women had a curable STI. The prevalence was 14.9% for *C*. *trachomatis* (95% CI:10.2–20.5), 1.0% for *N*. *gonorrhoeae* (95% CI: 0.1–3.5), 7.4% for *T*. *vaginalis* (95% CI:4.2–12.0), 19.3% for BV (95% CI: 14.1–25.4) and 2.5% for genital ulcers (95% CI: 0.8–5.7). Predictors for infection with curable STIs included women with a genital ulcer (adjusted odds ratio (AOR) = 35.0, 95% CI: 2.7–461.6) compared to women without a genital ulcer, women who used water for cleaning after visiting the toilet compared to those who used toilet paper or other solid means (AOR = 4.1, 95% CI:1.5–11.3), women who reported having sexual debut ≤ 17 years compared to women having sexual debut ≥18 years (AOR = 2.7, 95% CI:1.1–6.6), and BV-positive women (AOR = 2.7, 95% CI:1.1–6.6) compared to BV-negative women.

**Conclusion:**

One in five women attending ANC had a curable STI. These infections were associated with genital ulcers, hygiene practices, early sexual debut and bacterial vaginosis.

## Introduction

Sexually transmitted infections (STIs) are a major public health concern globally and especially among women in resource-limited countries [1]. Common curable STIs among women in sub-Saharan Africa (sSA) include *Chlamydia trachomatis*, *Neisseria gonorrhoeae* and *Trichomona*s *vaginalis* [2]. Additionally, bacterial vaginosis (BV), which has been associated with an increased risk of incident infection with *C*. *trachomatis* and *N*. *gonorrhoeae* [3, 4], is highly prevalent [5]. These curable STIs and BV have been associated with a number of adverse pregnancy outcomes such as spontaneous abortion, ectopic pregnancy, preterm delivery, low birth weight, stillbirth, postpartum sepsis, and congenital infection [6]. Infants born of mothers with genital *C*. *trachomatis* are at an increased risk of suffering from neonatal pneumonia and ophthalmia neonatorum [7]. At present the efficacy of neonatal ocular prophylaxis with erythromycin for prevention of *C*. *trachomatis* ophthalmia is not clear [8]. These associated sequelae of STIs hence necessitate prevention and management of curable STIs and BV, especially during pregnancy.

Pregnant women attending antenatal care (ANC) in resource-limited countries are frequently screened for syphilis [9] and HIV [10], but rarely for other curable STIs or BV. Moreover, curable STIs and BV are associated with increased acquisition and transmission of HIV [11–13]. Among pregnant HIV positive women *C*. *trachomatis* is associated with increased risk of HIV mother-to-child transmission [14]. Many resource-limited countries have adopted the World Health Organization’s syndromic approach for the management of curable STI [15]. This syndromic approach is deficient in both sensitivity and specificity because STIs may be asymptomatic or have non-specific clinical signs and symptoms [16, 17].

There is a paucity of epidemiological and risk factor data on curable STIs among women residing in rural areas, including among pregnant women in coastal Kenya. Most of the recent information about the prevalence of curable STIs among women in Kenya has been from studies done among high-risk individuals [18], women residing in cities [19, 20] or from regions with high incidence of HIV [13]. Consequently, we assessed the prevalence and predictors of curable STIs in pregnant women attending ANC at a rural county hospital in Coastal Kenya.

## Methods

This was a cross-sectional study conducted from July to September 2015, at the ANC clinic of the Kilifi County Hospital (KCH), Kenya, a clinic attended to by approximately 4,000 women annually [21]. We aimed to enroll a convenience sample of 350 consecutive pregnant women to identify 20–25 women with *T*. *vaginalis* for detailed molecular studies of *T*. *vaginalis* and assessment of the vaginal microbiome. Here, we report on a sub-study of 202 women who were tested for curable STIs, i.e. *C*. *trachomatis*, *N*. *gonorrhoeae*, *T*. *vaginalis*, and for BV.

### Procedures

Recruitment: A nurse at the ANC clinic provided general information about the study to women who had completed their ANC visit. Women interested to know more about the study were invited to participate and up to nine consecutive women were enrolled per day.

Inclusion criteria: Women were eligible if they met the following criteria: age 18–45 years, gestation ≥ 14 weeks, resident of the Kilifi Health and Demographic Surveillance area [22] and willing to undergo free STI and BV screening procedures.

Study procedures: Consenting women underwent a short private interview with the nurse at the ANC clinic using a structured questionnaire capturing symptoms of vaginal infections. This was followed by a collection of vaginal swabs by the ANC nurse. During the collection of vaginal swabs, the nurse observed for abnormal vaginal discharge and genital ulcers. Three vaginal swabs were collected, by inserting one swab at a time approximately 2 inches into the vaginal opening and gently turning around twice ensuring rubbing the swab against the vaginal wall.

The first swab was used for BV diagnosis and vaginal smears were prepared by rolling the swab onto a microscope glass slide. Slides were air-dried at the clinic before being transported to the laboratory for heat fixation followed by Gram staining and microscopy using the scoring system described by Nugent [23]. Women who self-reported discharge and had a Nugent score of 7–10 were considered BV-positive, and were treated as recommended [8]. Women with a Nugent score of 7–10 but without discharge were considered BV-negative.

The second swab was used to diagnose *T*. *vaginalis* using a commercially available *T*. *vaginalis* InPouch^TM^ system (BioMed Diagnostics, White City, Oregon, USA). The vaginal swab was inoculated in the upper-chamber of the InPouch at the clinic. The inoculated InPouch was transferred to the laboratory for direct microscopy of the upper chamber, after which the upper chamber was merged with the lower chamber and incubated at 37°C. Daily microscopy was performed and samples with motile trichomonads within 5 days of culture were considered positive for *T*. *vaginalis*.

The third swab was placed in a sterile labeled 2 ml Eppendorf tube, the swab shaft was broken by bending the shaft against the neck of the Eppendorf tube. The bottom portion of the swab with the specimen was transported to the laboratory where it was immediately stored at -80°C for molecular studies of the vaginal microbiome. No transport or freezing medium was added.

Women provided fresh first catch urine for *C*. *trachomatis* and *N*. *gonorrhoeae* testing, that was performed by the GeneXpert^®^ CT/NG Assay (Cepheid, Sunnyvale, California), according to the manufacturer’s instructions.

Results for *C*. *trachomatis*, *N*. *gonorrhoeae*, for *T*. *vaginalis* direct microscopy and for BV were reported on the same day of the visit, and STI and BV treatment was offered according to Kenyan national guidelines [24], except for treatment of *N*. *gonorrhoeae* where ceftriaxone was used in place of quinolones [25]. Women who were negative on *T*. *vaginalis* direct microscopy but positive on culture were contacted to return to the ANC clinic for treatment immediately after the culture turned positive. Treatment was also offered for the partners of the women with curable STIs.

Syphilis and HIV test results were obtained from each participant’s ANC records. Syphilis results were based on testing for treponemal antibodies only, done at the hospital laboratory using a commercial test kit Advanced Quality One Step Anti-TP (InTec products INC, Xiamen, China). Rapid HIV testing was done by the Alere Determine HIV-1/2 kit (Alere Medical Co Ltd, Chiba, Japan), the First response HIV 1-2-0 kit (Premier Medical Corporation Limited, Daman, India) was used to confirm positive results, and the Uni-Gold kit (Trinity Biotech, Wicklow, Ireland) was used to resolve any discordant test results.

After clinical procedures were completed, all study participants were interviewed. The interviews were conducted in Kiswahili by a trained female fieldworker. The questionnaire covered sociodemographic, hygienic and sexual behavior questions. These included age, education level, marital status, number of children, occupation, consumption of alcohol, consumption of tobacco and other substances, age at first sex, number of sexual partners, history of STIs, and hygiene and sanitary practices.

### Ethical considerations

The study was approved by the KEMRI Scientific and Ethics Review Unit (#3022). All participants provided written informed consent for study participation. Permission to use hospital records on syphilis and HIV status was obtained from Kilifi County Health department.

### Data analysis

Data were entered in the REDCap^TM^ electronic data capture tool, version 6.5.0 (Vanderbilt University, Nashville, Tennessee). Prevalence of STIs was determined as the number of participants with either *C*. *trachomatis*, *N*. *gonorrhoeae* and/or *T*. *vaginalis* out of 202 women that could be tested, expressed as a percentage. Exact 95% binomial confidence intervals (CIs) were calculated for prevalence estimates. Bivariable logistic regression was used to determine associations of sociodemographic, hygienic, behavioral and clinical characteristics with curable STIs. Variables significant at p≤ 0.1 in bivariable analysis were entered into a multivariable model to identify independent associations. Crude odds ratios (COR) and adjusted odds ratios (AOR) were calculated. These statistical analyses were done using STATA version 13.1 (StataCorp, College Station, Texas).

## Results

**Fig 1:**
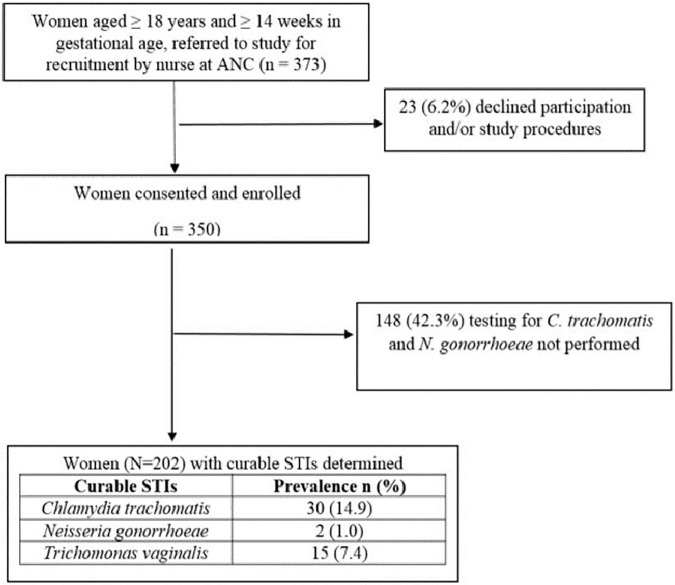
Flow chart of women attending antenatal care clinic at Kilifi County Hospital invited to participate in the study, June- September 2015

From June to September 2015, a total of 373 women were invited to participate in the study. Twenty-three (6.2%) women declined to participate citing no interest in research, lacking time or having no permission from spouse/partner as the main reasons. Of the 350 women enrolled, 148 (42.3%) women did not have *C*. *trachomatis* and *N*. *gonorrhoeae* tests performed due the unavailability of the tests at the beginning of the study. Therefore, a total of 202 women were tested for *C*. *trachomatis*, *N*. *gonorrhoeae*, *T*. *vaginalis* and BV and were included in this sub-analysis (Fig 1).

### Characteristics of the study population

The median age and gestation age including the interquartile range (IQR) for the participants was 26 (22–31) years and 24 (20–28) weeks respectively. The majority of the participants were married (93.6%) and Christian (72.3%). A quarter of the participants had secondary school education and above. The median age and IQR of sexual debut was 18 (16–20) years, although 20.8% of the participants were not sure or did not respond to this question. Forty-two percent of the participants had given birth before and the number of children ranged from 1–7. Sixty-two percent of the participants had previously experienced signs/symptoms associated with reproductive tract infection and one third had ever received syndromic treatment for genital signs or symptoms of infection. The socio-demographic characteristics of the participants are summarized in Table 1.

**Table 1:** Socio-demographic, hygienic, and behavioral characteristics of 202 women attending antenatal care and tested for sexually transmitted infections and bacterial vaginosis in Kilifi County Hospital, Kenya, July-September 2015.

	Total n = 202	Positive for curable STIs n = 42 (%)
***Demographic characteristics***		
**Age group (Years)**		
18–24	82	24 (29.3)
≥ 25	120	18 (15.0)
**Religion**		
Christian	146	28(19.2)
Muslim	28	7 (25.0)
Other/None	28	7 (25.0)
**Education**		
None	35	7 (20.0)
Primary	118	26 (22.0)
Secondary/Tertiary	49	9 (18.4)
**Marital status**		
Single	13	5 (38.5)
Married	189	37 (19.6)
**Residency**		
Living with partner	138	27 (19.6)
Not living with the partner	64	15 (23.4)
**Employment status**		
Employed/self-employed	115	20 (17.4)
Unemployed	87	22 (25.3)
**Parity**		
0	58	15 (25.9)
1–2	69	17 (24.6)
3+	75	10 (13.3)
Gestational age (weeks)**		
14–25	114	24 (21.1)
≥ 26	87	18 (20.7)
***Hygiene characteristics***		
**Toilet type**		
Flushing toilet	75	15 (20.0)
Pit latrine	105	22 (21.0)
Bush/Other	22	5 (22.7)
**Mode of cleaning after visiting the toilet**		
Tissue paper/other solid materials	64	7 (10.9)
Water	138	35 (25.4)
**Vaginal washing when bathing ǂ**		
External	32	6 (18.8)
Internal	166	35 (21.1)
***Behavioral characteristics***		
**Sexual debut age (years)**		
≤ 17	64	21 (32.8)
≥ 18	96	14 (14.6)
Don't know/no response	42	7 (16.7)
**Number of lifetime sex partners**		
1	96	16 (16.7)
2+	106	26 (24.5)
**Partner had other sexual partners in the last 6 months**		
No	182	38 (20.9)
Yes	20	4 (20.0)
**Alcohol consumption ever**		
No	160	35 (21.9)
Yes	42	7 (16.7)
**Tobacco smoking/chewing ever**		
No	191	41 (21.5)
Yes	11	1 (9.1)
**Drugs/substance use ever**		
No	194	39 (20.1)
Yes	8	3 (37.5)
***Sexually transmitted infections/ reproductive tract infection***		
**HIV**		
Negative	189	39 (20.6)
Positive	13	3 (23.1)
Syphilis***		
Negative	198	42 (21.2)
Positive	2	0 (0.0)
Bacterial vaginosis*		
Negative	162	29 (17.9)
Positive	39	13 (33.3)
***Clinical signs and symptoms of STI***^¥^		
**Previous history of vaginal discharge**		
No	75	13 (17.3)
Yes	127	29 (22.8)
**Previous syndromic treatment of genital infection**		
No	136	28 (20.6)
Yes	66	14 (21.2)
**Current vaginal discharge (self-reported)**		
No	52	10 (19.2)
Yes	149	32 (21.5)
**Abnormal vaginal discharge foul smell/color (observed)**		
No	165	32 (19.4)
Yes	36	10 (27.8)
**Dysuria**		
No	159	32 (20.1)
Yes	42	10 (23.8)
**Dyspareunia**		
No	150	29 (19.3)
Yes	51	13 (25.5)
**Vaginal itching**		
No	146	31 (21.2)
Yes	55	11 (20.0)
**Lower abdominal pain**		
No	107	24 (22.4)
Yes	94	18 (19.1)
**Genital warts**		
No	196	40 (20.4)
Yes	5	2(40.0)
**Genital ulcer (observed)**		
No	196	38 (19.4)
Yes	5	4 (80.0)
**Vaginitis**		
No	195	40 (20.5)
Yes	6	2 (33.3)

Curable STIs = Infection with any of the following sexually transmitted infectious agents: *Chlamydia trachomatis*, *Neisseria gonorrheae* and/or *Trichomonas vaginalis*

ǂ Four participants did not respond to this question

*Bacterial vaginosis result for one participant was not available

** Gestational age for one participant was missing

*** Syphilis hospital records for two participants were unavailable.

^¥^Clinical signs and symptoms of STI records for one participant were missing.

### Prevalence of curable STIs, BV, HIV and syphilis

A total of 20.8% (42/202) (95% confidence interval (CI): 15.4–27.0) women had a curable STI and 19.4% (39/202) (95% CI: 14.2–25.6) women had BV i.e. Nugent score of 7–10 and reported vaginal discharge. The prevalence was 14.9% for *C*. *trachomatis* (95% CI: 10.2–20.5), 1.0% for *N*. *gonorrhoeae* (95% CI: 0.1–3.5) and 7.4% for *T*. *vaginalis* (95% CI: 4.2–12.0) (Fig 1). Five (11.9%) women had more than one STI, while 13 (31%) out of the 42 women with a curable STI also had BV. Only two women (1.0%) had asymptomatic BV and none of them had a curable STI (data not shown).

Thirteen women were HIV-positive, a prevalence of 6.4% (95% CI: 3.4–10.8), including four (2.0%; 95% CI: 0.5–5.0) women who were newly diagnosed. Of note, HIV prevalence in 82 women aged 18–24 vs. 120 women aged ≥ 25 years was 0% vs. 10.8% (p = 0.002). All nine HIV positive women who knew their status were on anti-retroviral therapy. Among the 13 women with HIV, three women had curable STIs, i.e. a prevalence of 23.1% (95% CI: 5.0–53.8). All women with HIV and a curable STI also had BV. The prevalence of curable STIs, or BV in the 13 women with HIV was not statistically significantly different from that of the 189 women without HIV (data not shown).

Two women had treponemal antibodies, a prevalence of 1.0% (95% CI: 0.1–3.6). Prevalence of HIV, treponemal antibodies and *T*. *vaginalis* for the 148 women who were not tested for *C*. *trachomatis* and *N*. *gonorrhoeae* was not statistically significantly different than the prevalence of HIV, syphilis, and *T*. *vaginalis* in the 202 tested for *C*. *trachomatis* and *N*. *gonorrhoeae* in the sub-study.

### Clinical signs and symptoms

The most commonly self-reported symptoms amongst all participants were vaginal discharge (73.8%) and lower abdominal pain (46.5%). However, during collection of specimen by the study nurse, only 17.3% of the participants had an abnormal discharge (defined as excess discharge/foul smelling discharge/colored discharge) upon examination. Five (2.5%) women had genital ulcers upon examination, of which 4 (9.6%) of the 42 women with a curable STI vs. 1 (0.6%) of the 159 women without a curable STI (p = 0.001). None of the women with genital ulcers had HIV or treponemal antibodies. Other self-reported clinical signs or symptoms were not associated with curable STIs.

Based on symptoms routinely used in syndromic management of STIs (i.e. genital ulcer, lower abdominal pain or abnormal vaginal discharge), 45.2% of the 42 women with curable STIs were asymptomatic.

### Risk factors/predictors

In bivariable analysis, curable STIs were more common among participants who were ≤24 years, had less than 3 children, had a sexual debut ≤17 years, used water to clean themselves after visiting the toilet, had a genital ulcer or were BV positive (Table 2).

**Table 2:** Bivariable and multivariable analysis of socio-demographic, hygienic, behavioral, and clinical characteristics of pregnant women with curable sexually transmitted infections attending antenatal clinic at Kilifi County Hospital, July—September 2015 (n = 202).

	CurableSTIs n(%)	Bivariable analysis			Multivariable analysis	
Demographic characteristics		COR (95% C.I)		P- value	AOR (95% C.I)	P-value
**Age group (Years)**						
18–24	24 (29.3)	2.3 (1.2–4.7)	**0.016**		1.8 (0.7–4.4)	0.226
≥ 25	18 (15.0)	Ref				
**Religion**						
Christian	28 (19.2)	Ref				
Muslim	7 (25.0)	1.4 (0.5–3.6)	0.483		-	-
Other/None	7 (25.0)	1.4 (0.5–3.6)	0.483		-	-
**Education**						
None	7 (20.0)	Ref				
Primary	26 (22.0)	1.1 (0.4–2.9)	0.797		-	-
Secondary/Tertiary	9 (18.4)	0.9 (0.3–2.7)	0.851		-	-
**Marital status**						
Single	5 (38.5)	Ref				
Married	37 (19.6)	0.4 (0.1–1.3)	0.115		-	-
**Residency**						
Living with partner	27 (19.6)	Ref				
Not living with partner	15 (23.4)	1.3 (0.6–2.6)	0.529		-	-
**Employment status**						
Employed/self-employed	20 (17.4)	Ref				
Unemployed	22 (25.3)	1.6 (0.8–3.2)	0.173		-	-
**Parity**						
0	15 (25.9)	2.3 (0.9–5.5)	**0.071**		2.7 (0.8–8.9)	0.111
1–2	17 (24.6)	2.1 (0.9–5.0)	**0.087**		1.9 (0.7–5.2)	0.237
3+	10 (13.3)	Ref				
**Gestational age (weeks)**						
14–25	23 (20.5)	Ref				
≥ 26	18 (20.7)	1.0 (0.5–2.0)	0.979		-	-
**Hygiene characteristics**						
**Toilet type**						
Flushing toilet	15 (20.0)	Ref				
Pit latrine	22 (21.0)	1.1 (0.5–2.2)	0.876		-	-
Bush/Other	5 (22.7)	1.2 (0.4–3.7)	0.781		-	-
**Mode of cleaning after visiting the toilet**						
Tissue paper/other solid material	7 (10.9)	Ref				
Water	35 (25.4)	2.8 (1.2–6.6)	**0.022**		4.1 (1.5–11.3)	**0.007**
Vaginal washing when bathing*						
External	6 (18.8)	Ref				
Internal	35 (21.1)	1.2 (0.4–3.0)	0.766		-	-
**Behavioral characteristics**						
**Sexual debut age (years)**						
≤ 17	21 (32.8)	2.9 (1.3–6.2)	**0.007**		2.7 (1.1–6.6)	**0.026**
≥ 18	14 (14.6)	Ref				
Don't know/no response	7 (16.7)	1.2 (0.4–3.2)	0.754		-	-
**Number of lifetime sex partners**						
1	16 (16.7)	Ref				
≥2	26 (24.5)	1.6 (0.8–3.3)	0.171		-	-
**Partner has had other sexual partner(s) in the last 6 months**						
No	38 (20.9)	1.1 (0.3–3.3)	0.927		-	-
Yes	4 (20.0)	Ref				
**Alcohol consumption ever**						
No	35 (21.9)	1.4 (0.6–3.4)	0.461		-	-
Yes	7 (16.7)	Ref				
**Tobacco smoking/chewing ever**						
No	41 (21.5)	2.7 (0.3–22.0)	0.344		-	-
Yes	1 (9.1)	Ref				
**Drugs/Substance use ever**						
No	39 (20.1)	0.4 (0.1–1.8)	0.248		-	-
Yes	3 (37.5)	Ref				
***Clinical characteristics***						
**HIV status**						
Negative	39 (20.6)	Ref				
Positive	3 (23.1)	1.2 (0.3–4.4)	0.834		-	-
**Bacterial vaginosis**						
Negative	29 (17.9)	Ref				
Positive	13 (33.3)	2.3 (1.1–5.0)	**0.036**		2.7 (1.1–6.6)	**0.031**
**Clinical signs and symptoms of STI**						
**Previous history of vaginal discharge**						
No	13 (17.3)	Ref				
Yes	29 (22.8)	1.4(0.7–2.9)	0.353		-	**-**
**Previous syndromic treatment of genital infection**						
No	28 (20.5)	Ref				
Yes	14 (21.2)	1.0 (0.5–2.1)	0.918		-	**-**
**Current vaginal discharge (self-reported)**						
No	10 (19.2)	Ref				
Yes	32 (21.5)	1.1 (0.5–2.5)	0.732		-	**-**
**Abnormal vaginal discharge (observed)**						
No	32 (19.4)	Ref				
Yes	10 (27.8)	1.6 (0.7–3.6)	0.265		-	**-**
**Dysuria**						
No	32 (20.1)	Ref				
Yes	10 (23.8)	1.2 (0.6–2.8)	0.602		-	**-**
**Dyspareunia**						
No	29 (19.3)	Ref				
Yes	13 (25.5)	1.4 (0.7–3.0)	0.352		-	**-**
**Vaginal itching**						
No	31 (21.2)	Ref				
Yes	11 (20.0)	0.9 (0.4–2.0)	0.848		-	**-**
**Lower abdominal pain**						
No	24 (22.4)	Ref				
Yes	18 (19.1)	0.8 (0.4–1.6)	0.568		-	**-**
**Genital warts**						
No	40 (20.4)	Ref				
Yes	2 (40.0)	2.6 (0.4–16.1)	0.304		-	**-**
**Genital ulcer (observed)**						
No	38 (19.4)	Ref				
Yes	4 (80.0)	16.6 (1.8–153.1)	**0.013**		35.0 (2.7–461.6)	**0.031**
**Vaginitis**						
No	40 (20.5)	Ref				
Yes	2 (33.3)	1.9 (0.3–11.0)	0.454		-	**-**

Curable STI = Infection with any of the following sexually transmitted infectious agents: *Chlamydia trachomatis*, *Neisseria gonorrheae*, *Trichomonas vaginalis*

AOR: Adjusted Odds Ratio

CI: Confidence interval

COR: Crude Odds Ratio

*One participant did not respond to this question

In multivariable analysis, independent predictors associated with curable STIs included women with a genital ulcer (AOR = 35.0, 95% CI: 2.7–461.6) compared to women without a genital ulcer, women who used water for cleaning after visiting the toilet compared to those who used toilet paper or other solid materials (AOR = 4.1, 95% CI: 1.5–11.3), women who reported having sexual debut ≤17 years compared to women having sexual debut ≥18 years (AOR = 2.7, 95% CI: 1.1–6.6), and BV-positive women (AOR = 2.7, 95% CI: 1.1–6.6) compared to BV-negative women (Table 2).

## Discussion

We found that one in five out of 202 pregnant women attending the ANC at the Kilifi County Hospital, Kenya, during the period July to September 2015 had at least one curable STI. The high burden of curable STIs was mostly due to cases of *C*. *trachomatis* (14.9% of women) and *T*. *vaginalis* (7.4% of women). We also found approximately 19.4% of the women to have BV, defined as the presence of vaginal discharge in combination with a Nugent score ≥7. Four out of the 5 women with genital ulcers also had a curable STI. Our study documented that having genital ulcers, cleaning with water as opposed to use of toilet paper after visiting the toilet, early sexual debut, and BV were strongly associated with the presence of curable STIs.

The high prevalence (14.9%) of *C*. *trachomatis* found in this study is comparable to a prevalence of 13% (95% CI: 8.6–16.8) among women seeking family planning services in Nairobi, Kenya [20] and 9.9% (95% CI: 7.2–13.2) among women in a multi-country (Kenya, Rwanda and South Africa) cross-sectional study [26]. Two other recent studies reported lower prevalence rates of *C*. *trachomatis*: 5.6% (95% CI: 4.4–7.0) among 1300 HIV-seronegative ANC-attending women at two district hospitals in rural western Kenya [13], and 6.0% (95% CI: 3.6–9.3) in another study among 300 women attending outpatient clinics in Nairobi [27]. Since the high prevalence of *C*. *trachomatis* among pregnant women may likely reflect ongoing transmission of *C*. *trachomatis* among the general rural population in Kilifi, further studies confirming the high prevalence and ongoing *C*. *trachomatis* transmission among this population would be helpful. *Chlamydia* infection has been found to be an independent predictor of HIV-1 acquisition in a recent study from western Kenya, with also syphilis, yeast infection/colonisation and BV showing strong associations with incident HIV-1 infection [13].

We documented a prevalence of *T*. *vaginalis* (7.4%) comparable to 6.0% among HIV-seronegative ANC-attending women in rural western Kenya [13], and 7.7% among women attending a child-health clinic in Mombasa, Kenya [28]. In vitro evidence suggests that *T*. *vaginalis* may alter the vaginal microbiome towards BV [29]. *T*. *vaginalis* has also been associated with increased viral shedding and increased acquisition of HIV [30, 31], potentially contributing to a significant number of additional HIV infections globally [31].

The prevalence of HIV-1 (6.4%) in this study was substantial, with all known and newly diagnosed cases occurring amongst the 120 women ≥25 years. In Coastal Kenya, HIV-1 prevalence estimates in women aged 15–24 years decreased from 5.7% in 2007 to 2.0% in 2012 (p = 0.026) [32]. Our results suggest a further decline in HIV-1 prevalence, among women ≤ 24 years.

A relatively low prevalence of *N*. *gonorrhoeae* (1%) and treponemal antibodies (1%) were found in our study. The prevalence of treponemal antibodies was similar to the study among HIV-seronegative ANC-attending women in rural western Kenya, [13] while the *N*. *gonorrhoeae* prevalence in the latter study was 3.0%.

BV diagnosis in our study was based on an *a priori* self-report of vaginal discharge in combination with a Nugent score of ≥7 (while only 2 (1%) women had asymptomatic BV). Women with BV had an almost 3-fold higher risk of infection with a curable STIs than women without BV, comparable to other studies [4, 33]. It might be advisable to better document the presence of BV. However, general screening for BV among all women attending ANC, using the Nugent score [23] or Amsel's criteria [34], would require significant commitment of resources and staff. Using simple point of care testing, Madhivanan *et al*. [35] demonstrated that a vaginal pH >4.5 and a positive whiff test had a sensitivity of 83% but a specificity of only 47% for diagnosing BV. Although, not specific this approach may be adopted for BV screening in resource poor settings. Alternatively, presumptive treatment with intravaginal metronidazole (750 mg) plus miconazole (200 mg) for five consecutive nights each month for 12 months was shown to be effective to improve vaginal health and to decrease susceptibility to bacterial STIs, in a recent trial among non-pregnant women [36], but the frequency of treatment would make it challenging to upscale this intervention.

Our findings reinforce the need to integrate STI services at ANC. Screening for *T*. *vaginalis* can be achieved at a relatively affordable cost (US $ 5), making screening for *T*. *vaginalis* potentially feasible. Recent acquisition by the Kenyan government of over 120 GeneXpert IV machines for county and sub-county hospitals for tuberculosis testing [37], has potentially expanded the diagnostic capacity for curable STIs using the same testing platform at larger hospitals. However, the cost of the *C*. *trachomatis* and *N*. *gonorrhoeae* test kits are currently high. Without assay costs becoming considerably less expensive, it seems unlikely that ANC-attending women will be routinely tested for *C*. *trachomatis* and *N*. *gonorrhoeae*. Cost-effective studies have played an important role of informing policy on the gains of syphilis screening in pregnancy while minimizing on the expenditure [38]. Such cost-effectiveness studies of ANC screening and treatment of *C*. *trachomatis*, *N*. *gonorrhoeae*, and *T*. *vaginalis* should be conducted in resource poor settings.

Treatment of STIs in both infected persons and their partner is pivotal for STI management, ensuring reduction in cases of reinfection or non-resolving infections and prevention of adverse outcomes [8]. In our study a presumptive patient delivered partner treatment approach was adopted. Despite the fact that we did not collect information on the number of partners who were treated, this approach has been shown to be acceptable and feasible among pregnant women in Kenya [39].

Early sexual debut was strongly associated with curable STIs in our study population, similar to findings in other studies [40, 41]. Kilifi county is one of the poorest counties in Kenya, characterized by low literacy levels mostly among women, young age at marriage of women, and high fertility rates [42]. Structural changes focusing on the economic empowerment of communities, including improved access to education, and legislation protecting women from early marriage will be necessary to reduce the high burden of curable STIs among pregnant women.

A salient predictor of curable STIs in our study population was the use of water to clean after visiting the toilet as compared to those women who reported to use toilet paper or other solid materials. Unfortunately, our hygiene question did not distinguish urinating from defecating, or inquired whether the cleaning mode included both anus and vagina. It is possible that such cleaning transported bacteria from the perianal area to the vagina, as pathogens causing curable STIs may be found in paragenital areas [43]. Moreover, there is a strong correspondence in bacterial species and loads between the vagina and rectum of pregnant women [44]. McClelland et.al [31] showed that vaginal washing increased risk of HIV-1 acquisition among African women. Although, we did not assess whether water used by our study participants was shared, sharing of water may potentially lead to contamination of the vagina [45]. Pereira-Neves and Benchimol [46] showed *in vitro* that *T*. *vaginalis* remained viable and infective in swimming pool water samples for several hours. Further research is required to investigate the potential role that sanitary and hygiene practices may play in women with high prevalence of curable STIs and BV.

Observed genital ulcers had the strongest association with curable STIs, and were found in 2.5% of study participants. Presumptively treating curable STIs on the basis of observed genital ulcers in pregnant women would have treated ~10% of the curable STIs in our study. Studies examining microbial aetiology of genital ulcer disease, have shown that genital ulcers can be attributed to several sexually transmitted pathogens with most cases being attributed to Herpes Simplex virus type 2 [47, 48]. Genital ulcers were also identified as the strongest predictor of acute HIV acquisition in attendees of STD clinics who initially had HIV-seronegative or discordant rapid test results [49]. While in our study no women with genital ulcers had HIV, observing genital ulcers in women may help initiate risk reduction during ANC counselling and targeted acute HIV screening [50].

Our study had some limitations. First, our study only included a relatively small sample of pregnant women limiting the precision of our prevalence estimates. Second, we only included adult women and were not able to enroll pregnant women who were minors. Minors are potentially more vulnerable and may have presented with different STIs. Third, we included only women residing within the Kilifi Health and Demographic Surveillance Area [22], and therefore will have excluded women from outside the surveillance area who may have different health characteristics. Fourth, our hygiene and sanitary questions were few, and limited [51].

Finally, this was a cross-sectional study, only allowing for an assessment of associations and we did not collect data on pregnancy outcomes.

Despite these limitations, we showed that there is high prevalence of curable STIs among pregnant women attending an antenatal clinic at Kilifi County Hospital, Kenya. Curable STIs were associated with genital ulcers, hygienic practices, early sexual debut and BV. We recommend that antenatal care programs consider strengthening their diagnostic screening for curable STIs and BV.
